# The impact of *Rhodiola rosea* on the gut microbial community of *Drosophila melanogaster*

**DOI:** 10.1186/s13099-018-0239-8

**Published:** 2018-03-20

**Authors:** Khachik E. Labachyan, Dara Kiani, Evgueni A. Sevrioukov, Samuel E. Schriner, Mahtab Jafari

**Affiliations:** Department of Pharmaceutical Sciences, University of California Irvine, Irvine, CA 92697 USA

**Keywords:** *Drosophila melanogaster*, *Rhodiola rosea*, *Acetobacter pomorum*, *Lactobacillus plantarum*, Bacterial load, Herbal extracts, Colony forming units, Quantitative RT-PCR, Disk diffusion, 16S rRNA gene sequencing

## Abstract

**Background:**

The root extract of *Rhodiola rosea* has historically been used in Europe and Asia as an adaptogen, and similar to ginseng and *Shisandra*, shown to display numerous health benefits in humans, such as decreasing fatigue and anxiety while improving mood, memory, and stamina. A similar extract in the *Rhodiola* family, *Rhodiola crenulata*, has previously been shown to confer positive effects on the gut homeostasis of the fruit fly, *Drosophila melanogaster.* Although, *R. rosea* has been shown to extend lifespan of many organisms such as fruit flies, worms and yeast, its anti-aging mechanism remains uncertain. Using *D. melanogaster* as our model system, the purpose of this work was to examine whether the anti-aging properties of *R. rosea* are due to its impact on the microbial composition of the fly gut.

**Results:**

*Rhodiola rosea* treatment significantly increased the abundance of *Acetobacter*, while subsequently decreasing the abundance of *Lactobacillales* of the fly gut at 10 and 40 days of age. Additionally, supplementation of the extract decreased the total culturable bacterial load of the fly gut, while increasing the overall quantifiable bacterial load. The extract did not display any antimicrobial activity when disk diffusion tests were performed on bacteria belonging to *Microbacterium*, *Bacillus*, and *Lactococcus*.

**Conclusions:**

Under standard and conventional rearing conditions, supplementation of *R. rosea* significantly alters the microbial community of the fly gut, but without any general antibacterial activity. Further studies should investigate whether *R. rosea* impacts the gut immunity across multiple animal models and ages.

**Electronic supplementary material:**

The online version of this article (10.1186/s13099-018-0239-8) contains supplementary material, which is available to authorized users.

## Background

*Rhodiola rosea*, informally referred to as the ‘golden root’ or ‘arctic root’, is an adaptogenic plant that has been reported to display positive effects on central nervous system activity and cardiovascular function [1–4]. The additional therapeutic effects of *R. rosea*, which derive primarily from its root extract, have been outlined in clinical trials for improving mental and physical work capacity during stress, alleviating mental distress, and ameliorating symptoms of depression [2, 5–10]. Although the traditional medicinal uses of *R. rosea* derive from Eastern Europe and Asia, *R. rosea* products have gained popularity worldwide among athletes as a natural remedy to prevent fatigue and improve performance [11]. We reported that *R. rosea* significantly extended both mean (24%, both sexes) and maximum (16% in males, 31% in females) lifespan of the fruit fly, *Drosophila melanogaster* [12, 13]. The lifespan extension properties of *R. rosea* appear to be conserved among model species since the plant has been shown to extend the lifespan of worm and yeast models as well [14, 15]. The mechanism of lifespan extension with *R. rosea*, however, remains to be determined.

*Drosophila melanogaster* is emerging as an important model to examine the interactions between non-pathogenic microbes within the host. Since *D. melanogaster* can be easily manipulated genetically and experimentally, it can serve as a good model to enhance our understanding of animal–microbial symbiosis. Utilizing the *Drosophila* model system provides an integrative approach to study the relationship between an herbal extract supplementation and the impact it may have on the gut microbial composition. Another species of the *Rhodiola* family, *Rhodiola crenulata*, also exhibits multiple pharmacological traits like that of *R. rosea*, such as stress protection, neuroprotection, high altitude sickness mitigation, and anti-inflammatory activity [16–19]. Moreover, *R. crenulata* has been demonstrated to treat metabolic disorders in rats [20] and increase intracellular antimicrobial peptide expression while improving gut morphology in fruit flies [21]. Here we suspect that *R. rosea* may act like *R. crenulata* in that it may change the microbial composition of *D. melanogaster.* Additionally, *R. rosea* may mimic numerous other herbal therapies that have been reported to alleviate gastrointestinal and metabolic disorders, which are particularly prevalent in the process of age-related microbial dysbiosis [22–24]. More specifically, the intestinal microbiota is significantly altered during severe age-related physiological ailments, such as obesity, insulin resistance, and general frailty, suggesting that age-related changes in the gut may have an impact on overall healthspan and lifespan [25–27].

The average adult *Drosophila* intestine harbors only 5–20 microbial species which primarily belong to the families *Enterobacteriaceae, Acetobacteraceae*, and the order *Lactobacillales* [28–31]. Of these three strains, the only bacterial order present in considerable amounts in both *Drosophila* and mammals is *Lactobacillales* [32–34]. When evaluating the microbial differences between fruit flies, it appears that the microbial content of *D. melanogaster*, independent of species uniformity, is similar between species that are fed on the same diet [35]. Conversely, more closely related species that feed on different diets are known to have a contrasting and diverse microbial compositions [35]. These findings suggest that certain bacterial families, such as *Acetobacteraceae*, may favor the low pH and high ethanol conditions present in fermenting fruits, thus influencing the microbiota of flies which favor fruit based diets [35].

The purpose of this work was to examine whether the anti-aging properties of *R. rosea* are due to its impact on the microbial composition of the fly gut. To date, there have been no published studies highlighting the impact of anti-aging botanical extracts on the microbial composition of the gut. These results will aim to build support for investigating the effects of botanical extracts on the gut microbiota and how they may help prevent against age-related intestinal diseases.

## Methods

### Fly strain and treatment

Oregon-R flies were obtained from the Bloomington *Drosophila* Stock Center at Indiana University. *Rhodiola rosea* extract (SHR-5) was obtained from the Swedish Herbal Institute. An independent HPLC analysis of this extract was performed by Alkemists Pharmaceuticals (Costa Mesa, CA) and this formulation was found to contain 1.7% salidroside and 4.5% total rosavins (data on file).

### Feeding

Food composition and detailed housing techniques are described in Schriner et al. and Jafari et al. [13, 36].

Flies used in these assays were raised from larvae (50–80 eggs per vial) in 5 mL of standard banana-molasses food composed of a 9% carbohydrate and a 3.6% yeast content. Upon hatching from pupae, flies were transferred to autoclaved jars at a density of 300 per jar (150 males and 150 females) and separated by treatment. *Rhodiola rosea* (25 mg/mL) was supplied to the adults by mixing with the yeast solution (4% yeast and 1% acetic acid) and was overlaid on top of the banana-molasses food while control flies only received the yeast solution with the food. 400 µL of both the yeast solution containing treatment as well as the non-treatment yeast solution was added on the food. Survivors were counted every 2 days and transferred to newly autoclaved jars. The dose of 25 mg/mL was used as the optimal concentration as this dose has consistently resulted in lifespan extension in both sexes [12]. Flies were maintained at 22 ± 1 °C under a 12 h light: 12 h dark cycle for all experiments.

### DNA extraction and qRT-PCR analysis

Flies were separated into six groups of five (n = 5), per treatment and sex, and surface sterilized in 75% ethanol, 10% bleach, and DPBS, all for 1 min each. Flies were then homogenized and placed in 500 µL of DPBS. DNA extractions were performed using the DNeasy Blood and Tissue Kit (Qiagen, West Sussex, UK) per manufacturer’s protocol but with the addition of 20 mg/mL lysozyme. The extracted DNA was stored at − 4 °C before qRT-PCR analysis. For estimation of *Lactobacillus plantarum*, *Acetobacter pomorum*, and 16S rDNA gene abundance, amplifications of each sample of extracted DNA was performed with each respective primer as described by Wong et al. [37]. The reaction mix comprised of 10 μL Power SYBR green PCR master mix (Applied Biosystems), 2 μL 10 μM primers (1 μL of each forward and reverse), 6 μL of sterile water, and 2 μL of approximately 25 ng DNA template in a 20 μL volume, with reagents being used as the negative controls. Amplifications were conducted in a Miniopticon (Bio-Rad) with the following thermal profile: 95 °C for 5 min, 40 amplification cycles of 95 °C for 15 s, 55.2 °C for 30 s, and 60 °C for 30 s, and a dissociation cycle of 95 °C for 15 s, 60 °C for 15 s, and then brought back to 95 °C. The average threshold cycle (Ct) values of two technical replicates per sample and primer set were calculated against a normalizing gene and quantification levels were calculated thereafter.

### Colony forming unit (CFU) analysis

Bacterial growth plates were generated according to the following recipes:

- *Lactobacili* MRS agar: 70 g/L of BD Difco *Lactobacili* MRS agar
- Nutrient agar: 5 g/L peptone, 3 g/L yeast, 15 g/L agar, 5 g/L NaCl.

All media were autoclaved at 121 °C for 15 min. Flies were separated into six groups of five (n = 5), per treatment and sex, and surface sterilized sequentially in 10% bleach solution for 1 min, 75% ethanol for 1 min, and PBS for 1 min. Flies were then homogenized in 500 µL of PBS. A series of dilutions were performed in order to have quantifiable number of colonies, which vary depending on the age of the fly. 50 µL of diluted fly homogenate was plated on each media and spread evenly. The plates were then incubated at 28 °C for 48–76 h. Plates were scanned with an Epson v600 scanner and analyzed with ImageJ [38].

### Antimicrobial assays

BD BBl Prepared Plated Media (Mueller–Hinton II Agar) was purchased from Fisher Scientific. Culturable bacteria isolated from both the environment, control, and treated flies were picked individually and grown overnight at 37 °C in Luria–Bertani broth media and then diluted until an OD600 measurement of 0.08–0.1 was observed, corresponding to a 0.5 McFarland standard and 1.5 × 10^8^ CFU/mL. The incubation temperature of 37 °C sufficiently promotes the growth of *Microbacterium*, *Bacillus*, and *Lactococcus*. The resulting media was plated on Mueller–Hinton II agar and then treated with 20 µL of *R. rosea*, Kanamycin (positive control), and DI water on disks of autoclaved Whatman filter paper #1. Concentrations of *R. rosea* used were 100, 50, 15, 10 and 1.5 mg/mL. The concentration of Kanamycin used was 1.5 mg/mL. Plates were incubated at 37 °C for another 24 h and then scanned with an Epson v600 scanner and analyzed with ImageJ [38]. The bacteria originally used for this assay was identified by 16S rRNA Sanger sequencing (GENEWIZ) and the resulting raw data chromatograms were visualized by using the Chromas Pro software (Technolysium Ltd.) and then identified with BLASTN (2.7.1 +) searches [39].

## 16S rRNA gene sequencing

All samples were DNA extracted, amplified, and sequenced by the Integrated Microbiome Resource lab (IMR) at Dalhousie University (Halifax, Canada). In brief, DNA was extracted from 5 mg of frozen flies per sample using the QIAamp PowerFecal DNA Kit (Qiagen) per manufacturer’s protocol. All DNA samples were amplified by PCR targeting the 16S rRNA gene sequence (regions V6–V8) as previously demonstrated [40], and libraries were prepared by following the guidelines provided by Illumina (San Diego, USA; Part #15044223, Rev. B). The amplified 16S rDNA fragments were then sequenced using the Illumina MiSeq platform by using the Microbiome Helper workflow [40]. Raw sequences were analyzed with QIIME (Quantitative Insight Into Microbial Ecology) and FastQC (v0.11.5) coupled with PEAR (v0.9.10) was used to evaluate raw reads, identify ambiguous reads, and stitch the reads together [41–43]. Chimeric DNA molecules were screened using VSEARCH (v1.11.1) and removed with the UCHIME algorithm [44, 45]. Open-reference OTU (Operational taxonomic units) picking was performed at 97% identity using SortMeRNA and SUMACLUST and reads were clustered against the Greengenes database [46–48]. Low confidence OTUs were removed with a 0.1% threshold and the final OTU table was normalized per sample using DESeq2 [49].

### Statistical analysis

Parametric unpaired t tests with Welch’s correction were used to display statistical and graphical representations of the qRT-PCR and CFU data using GraphPad Prism version 7.00 for Mac OS X, GraphPad Software, La Jolla California USA, http://www.graphpad.com. Box-and-whisker plots were created to display the 25th to 75th percentiles of the data sets with a line in the middle of the plot as the median. Minimum to maximum values are shown by the whiskers. The 16S rRNA amplicon sequencing data was analyzed by converting the QIIME derived BIOM OTU table to a format compatible with STAMP (v2.1.3) [50]. Box plots were generated through STAMP to show the median of the data as a line, the mean of the data as a star, the 25th and 75th percentiles of the data as the top and bottom of the box, and whiskers to indicate the minimum and maximum values within 1.5 * (75th–25th percentile) of the median. Data points outside of the whiskers are shown as crosses. The statistical hypothesis test used for these samples was a Welch’s t test with the Storey’s FDR multiple test correction at 0.05 to control the false discovery rate [51]. Heatmap plots were generated through STAMP alongside PCA plots to show the proportion of sequences assigned to each feature with the use of a dendrogram to cluster features and samples.

## Results

### *Rhodiola rosea* significantly alters the microbial composition

**Fig. 1:**
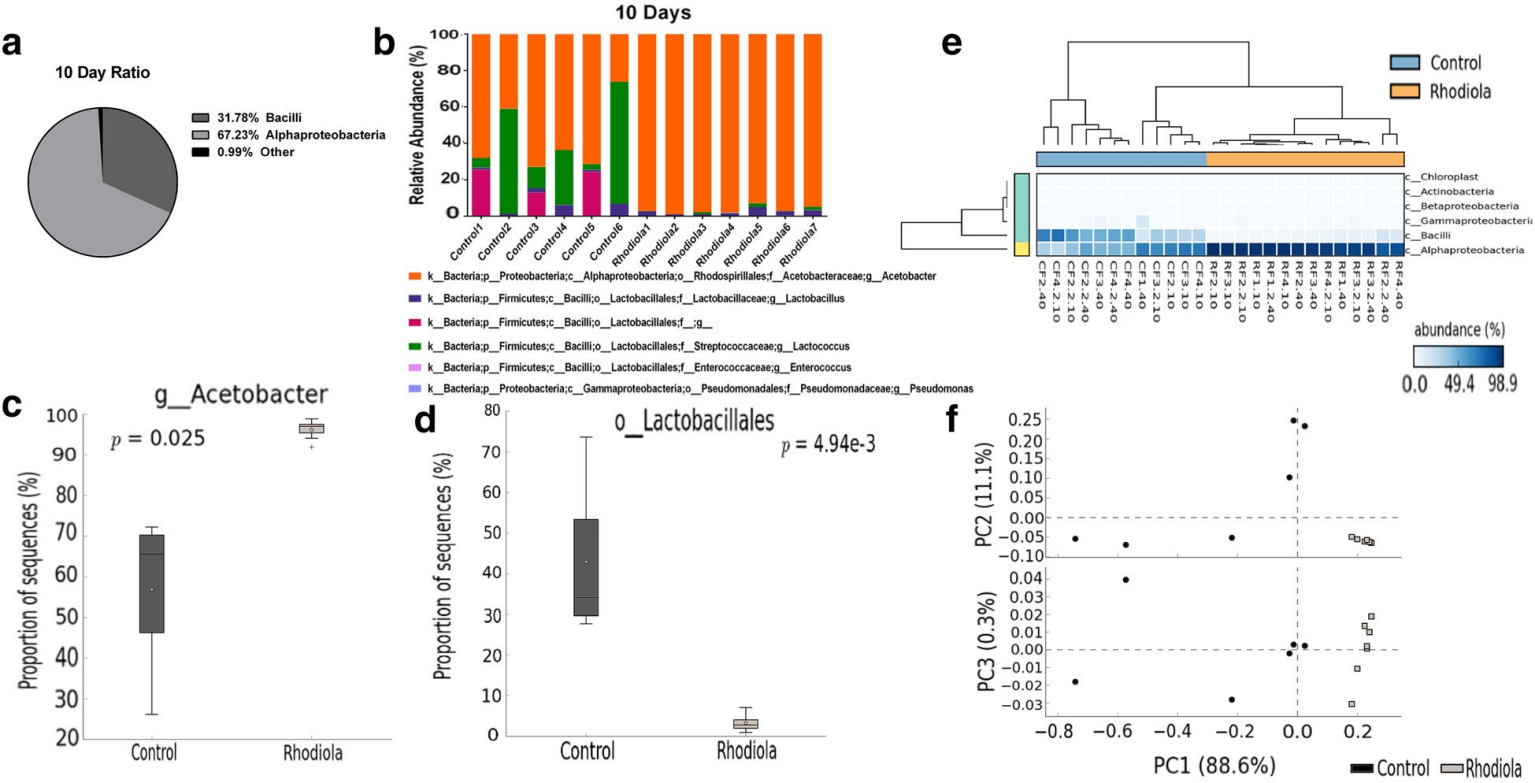
Populations within the female 10 days old *Drosophila* microbiota derived from 16S rRNA amplicon sequencing of the V6–V8 region. *CF* control group, *RF* treatment group. **a** The majority of the dominant bacteria in the flies belong to the classes *Bacilli* and *Alphaproteobacteria*. **b** Relative abundance of bacterial taxa. **c** Relative abundances of genus *Acetobacter* between control and treatment. **d** Relative abundances of order *Lactobacillales* between control and treatment. **e** Heatmap with a dendogram showing the abundance intensity of each sample relative to the class they belong to. **f** PCA plot showing the family level pattern of similarity between groups of each respective treatment

**Fig. 2:**
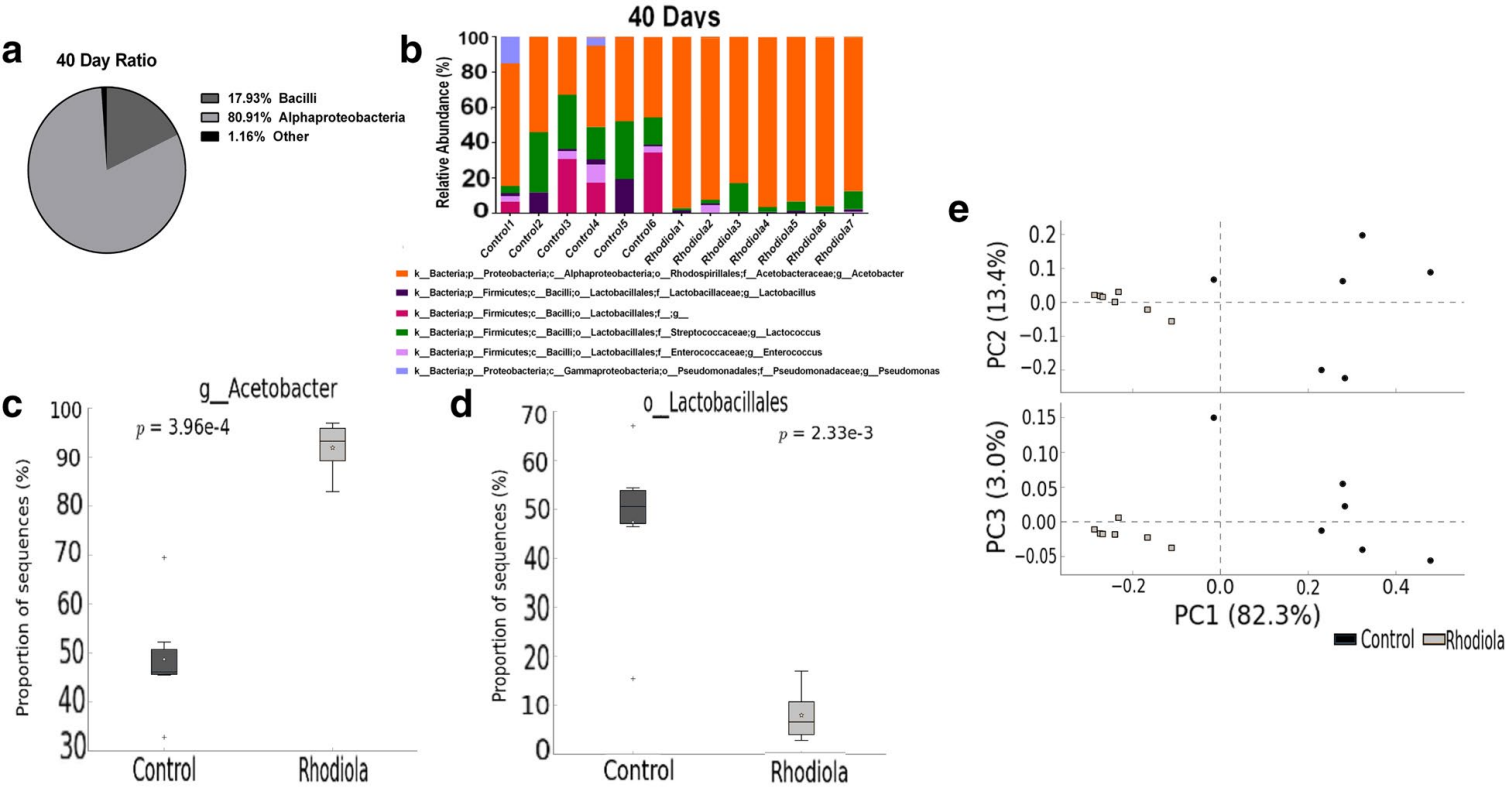
Populations within the female 40 days old *Drosophila* microbiota derived from 16S rRNA amplicon sequencing of the V6–V8 region. **a** The majority of the dominant bacteria in the flies belong to the classes *Bacilli* and *Alphaproteobacteria*. **b** Relative abundance of bacterial taxa. **c** Relative abundances of genus *Acetobacter* between control and treatment. **d** Relative abundances of order *Lactobacillales* between control and treatment. **e** PCA plot showing the family level pattern of similarity between groups of each respective treatment

The objective of this work was to study whether *R. rosea* changes the microbial composition of the fruit fly throughout its lifespan. After hatching from their pupae (Day 0), we housed the control flies and the *R. rosea* fed flies separately and started the experiment. The flies were placed on new media every other day and assayed at days 10 and 40, corresponding to ‘young’ and ‘old’ in respect to the fly strain used. The relative bacterial abundances generated from the flies were identified by 16S rRNA amplicon sequencing. It has been previously reported that the bacterial species which dominate the gut of young flies belong to the genus *Lactobacillus*, while the bacteria that dominate the gut of older flies belong to the genus *Acetobacter* [52]. However, our study revealed that classes *Bacilli* and *Alphaproteobacteria*, which include the genera *Lactobacillus* and *Acetobacter*, respectively, display different abundances of each class of bacteria at younger ages when compared to a prior study [52]. We observed an increased ratio of *Alphaproteobacteria* (67.23%) to *Bacilli* (31.78%) in 10 days old flies (Fig. 1a). In 40 days old flies, we observed a similar ratio, with *Alphaproteobacteria* (80.91%) dominating in abundance, displaying a trend that is similar to previous studies (Fig. 2a) [52].

When comparing *R. rosea* fed flies to control flies, the relative abundances of several individual bacterial taxa was differentially associated between each respective age group given treatment (Figs. 1b and 2b). Most of the taxa in the flies derived from the phyla *Firmicutes* and *Proteobacteria* (Figs. 1b and 2b). The presence of *Gammaproteobacteria*, which is an indication of dysbiosis in the fly gut [53], was present at no levels in the guts of 10 days old flies (Fig. 1b), but was present at minimal levels in only 2 samples (Control1 and Control4) in 40 days old flies (Fig. 2b). The genus *Lactococcus* (individual graph not shown) was more abundant in the guts of control flies for both time points (Figs. 1b and 2b), but only 40 days old flies given *R. rosea* treatment displayed significantly lower presence of this genus (p = 0.019, Welch’s t test with Storey FDR multiple test correction). The genus *Enterococcus* was minimally present in the guts of 40 days old flies (Fig. 2b), but showed no statistical significance when compared between control and treatment groups (p > 0.05, Welch’s t-test with Storey FDR multiple test correction).

The genus *Acetobacter* and the order *Lactobacillales* showed stark differences between control and treatment groups at both ages. At both 10 and 40 days, the levels of *Acetobacter* were significantly increased with *R. rosea* treatment, while levels of *Lactobacillales* were significantly decreased (Figs. 1c, d and 2c, d). A generated heatmap plot shows the intensity of abundance between control and *R. rosea* fed flies across various classes of bacteria (Fig. 1e). All control samples (indicated by a ‘C’ in front of the sample name) displayed abundance intensities for both *Bacilli* and *Alphaproteobacteria* classes, while all *R. rosea* samples (indicated by a ‘R’ in front of the sample name) displayed stronger abundance intensities for the class *Alphaproteobacteria* but lower intensities for the class *Bacilli*. Principal components analysis (PCA) plots were generated for both age groups by using the Euclidean distance as the dissimilarity metric to display the spatial variation of control and treatment groups across three principle axes (PC1, PC2, and PC3) (Figs. 1f and 2e).

### *Rhodiola rosea* alters the relative amounts of individual species abundance

**Fig. 3:**
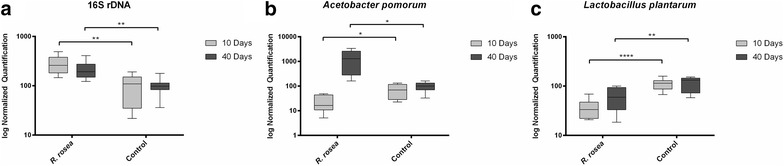
Real-time quantitative PCR results for 10 and 40 days old female flies. *p < 0.05; **p < 0.01. **a** 16S rDNA of the V3 hypervariable region and the species **b** *A. pomorum* and **c** *L. plantarum* showed statistically significant differences

In order to analyze the bacterial genera in more detail, quantitative real-time PCR (qRT-PCR) was utilized to evaluate the species level differences in our samples. Total bacterial content (measured through the 16S rDNA gene) as well as the relative abundances of two highly relevant bacterial species, *L. plantarum* and *A. pomorum*, were significantly altered with treatment of *R. rosea* (Fig. 3a–c). Total bacterial load was increased with treatment of *R. rosea* in females at both 10 and 40 days of age (Fig. 3a). 16S rDNA of the V3 hypervariable region showed significant differences between control and treatment with p values of 0.0026 and 0.0088 in 10 and 40 days, respectively (Fig. 3a). Additionally, *A. pomorum* was present at lower levels in *R. rosea* fed flies at 10 days of age, but present at higher levels with *R. rosea* supplementation at 40 days of age (Fig. 3b). *A. pomorum* differences between control and treatment resulted in p values of 0.0174 and 0.0178 in 10 and 40 days, respectively (Fig. 3b). Bacterial species belonging to *L. plantarum* were present at lower levels at both stages of the fly lifespan given treatment (Fig. 3c). *L. plantarum* differences between control and treatment resulted in p values of 0.0001 and 0.0074 in 10 and 40 days, respectively (Fig. 3c). Statistics were performed using the unpaired t test with Welch’s correction. Each group contained samples with n = 5 with four technical replicates each within 2 biological replicates.

### *Rhodiola rosea* decreases culturable bacterial load

**Fig. 4:**
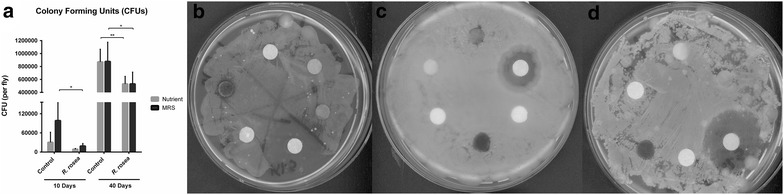
**a** Colony forming units of 10 and 40 days old female flies when plated on MRS and nutrient agar. *p < 0.05; **p < 0.01. Kirby-Bauer antimicrobial assays used to test genera **b** *Microbacterium*, **c** *Bacillus*, and **d** *Lactococcus* against *R. rosea* infused disks

To assess the amount of culturable bacterial load, homogenized flies were plated on both De Man, Rogosa and Sharpe (MRS) and nutrient agar. MRS agar has been established as the conventional bacterial media to cultivate microorganisms belonging to the *Lactobacillus* genus [54]. At earlier ages, the fly gut exhibits minimal colonization with microorganisms, thus resulting in a decreased bacterial load [30]. We showed here that the earlier stages of the fly life consisted of a lower amount of culturable bacteria, corresponding to less colony forming units (CFUs) (Fig. 4a). More importantly, *R. rosea* treatment significantly reduced the amount of CFUs in 10 days old flies plated on MRS media (Fig. 4a). Additionally, we observed that *R. rosea* decreased the amount of CFUs in 40 days old flies plated on both MRS and nutrient agar. The difference in CFUs between *R. rosea* and control fed flies at 40 days was more prominent in both media (30,000 CFUs) due to the exponential growth of bacteria that inhabit the later stages of the fly gut (Fig. 4a). Statistics were performed using the unpaired t test with Welch’s correction. For CFU testing, each group contained samples with n = 5 with six technical replicates each within one biological replicate.

### *Rhodiola rosea* does not have antimicrobial properties against *Microbacterium*, *Bacillus*, and *Lactococcus*

Multiple studies have demonstrated that plant extracts that exhibit zones of inhibition with diameters 10 mm may possess antimicrobial properties [55, 56]. *Rhodiola rosea* has previously been shown to exhibit antimicrobial activity on multiple strains of *Staphylococcus aureus*, but the source of growth (botanical garden in Poznań, Poland) and the composition of the extract (2.04% salidroside and 1.46% cinnamyl alcohol) were different than the *R. rosea* that was used in our study [57]. We performed Kirby–Bauer disk diffusion tests with *R. rosea* concentrations of 1.5, 15, 50, and 100 mg/mL with a positive control of Kanamycin (1500 µg/mL) and a negative control of DI water. The amount of total *R. rosea* extract added to each disk was 30, 300, 1000, and 2000 µg which corresponds to the concentrations above, with a total amount of 30 µg Kanamycin. We plated bacterial isolates grown in our lab belonging to the genera *Microbacterium* (Fig. 4b), *Bacillus* (Fig. 4c), and *Lactococcus* (Fig. 4d) against the previously mentioned concentrations of *R. rosea*. All concentrations of *R. rosea* appeared to not display zones of inhibition when plated across all three bacterial genera (Fig. 4b–d).

## Discussion

The aim of this study was to determine whether *R. rosea* can change the gut microbial community of *D. melanogaster*. Our group had previously reported that the root extract of *R. rosea* extends the lifespan and improves the healthspan of the fruit fly, but the exact underlying mechanisms of lifespan extension remains unclear [12, 13, 36]. In this study, we examined the impact of *R. rosea* on the microbial dynamics of the fly gut and whether changing the gut microbiome could be beneficial for host longevity. When evaluating the impact of *R. rosea* on the fly gut microbiota, we observed sex specific differences between fly groups which could be contributed to a variety of physiological factors. At adult stages, female fruit flies require a greater protein intake needed for egg production, thus consuming more environmental yeast when compared to their male counterparts [58]. Due to extensive contact with environmental nutrients, the female flies, along with their microbial communities, experience metabolism-related shifts through alteration of host signaling pathways [59]. Performing 16S rRNA sequencing exclusively on female fruit flies allowed for investigation into the environmental and nutrient microbe-altering effects of *R. rosea* and how it influenced the microbial community of the host. Studies involving both sexes and multiple strains of *Drosophila* will be required to thoroughly understand the paired effect of *R. rosea* and yeast consumption on the host microbiota.

Our results show that while control female Oregon-R fruit flies establish and maintain a consistent microbial composition throughout their lifespan, the *R. rosea* supplemented flies maintained a microbial composition which differed in relative abundance of order *Lactobacillales* and genus *Acetobacter* when compared to control (Figs. 1b and 2b). These changes, with respect to supplementation of *R. rosea*, are likely to vary between *Drosophila* strains, with additional factors influencing the microbiota such as the nutritional composition and sex [35, 60–62]. Male flies in our study displayed no significant changes in *L. plantarum*, *A. pomorum*, and the 16S rDNA gene when supplemented with *R.* rosea (p > 0.05, Unpaired Welch’s t test) (Additional file 1: Figure S1a–c). CFU tests revealed that male flies displayed significant decrease in CFU counts at early ages of their lifespan (p value = 0.0096 for MRS, p value = 0.0367 for nutrient), but no difference was observed at the later stages of their lifespan, where flies experience an increased bacterial load (Additional file 1: Figure S1d).

Our 16S rRNA amplicon sequencing identified the differences in diversity between control and *R. rosea* fed flies. In 10 days old flies, control flies had an average Operational Taxonomic Unit (OTU) count of 18.17, while *R. rosea* fed flies had a count of 16.14 (Additional file 2: Table S1). In 40 days old flies, control flies had a OTU count of 22.33, while *R. rosea* fed flies had a count of 17.7 (Additional file 2: Table S1). Although we observe a decrease in bacterial diversity in *R. rosea* fed flies, the total abundance of bacteria increase, as indicated through 16S rDNA qRT-PCR analysis (Fig. 3a). Although our results suggest that the *R. rosea* induced gut microbiome changes are age-dependent, to fully comprehend the time point where *R. rosea* begins to induce such changes, additional time points (i.e. time of eclosion) need to be evaluated. Furthermore, since several samples in our study missed certain bacterial genera (*Lactococcus*, *Enterococcus*), additional samples at various time points need to be evaluated to determine which microorganisms are natively present in the gut in comparison to which are acquired from the environment. To limit the impact on external inputs from contributing to the bacterial load within the *Drosophila* gut, many studies have utilized germ-free flies as a model to test the effect of individual bacteria on host physiology [63, 64]. Utilizing the gnotobiotic model will allow us to control the influence of environmental factors to discern how *R. rosea* directly affects individual bacterial species inside the host.

The most notable observations in this study resulted from the ability of *R. rosea* to increase the ratio of genus *Acetobacter* and decrease the order *Lactobacillales* at both the early and later stages of the fly lifespan (Figs. 1c, d and 2c, d). Observations were taken at the order level due to the presence of unidentified reads that belong to the families and genera under the *Lactobacillales* order. When comparing the 16S rRNA sequencing data with the 16S rDNA qRT-PCR reads, we noticed that although the genus *Acetobacter* was increased in 10 days old flies that were fed *R. rosea*, the species *A. pomorum* was significantly decreased in these flies (Figs. 1b and 3b). This contrast is possible due to the presence of other commensal species belonging to the genus *Acetobacter*, such as *Acetobacter pasteurianus*, *Acetobacter aceti,* and *Acetobacter tropicalis* [65]. We observed an opposite trend between treatment groups when comparing between the 16S rDNA amplification and CFU counts. A decrease in CFUs corresponded with an increase in 16S rDNA expression, indicating that *R. rosea* fed flies experience a lower culturable bacterial load but more overall bacteria (Figs. 3a and 4a). Interestingly enough, both MRS and nutrient agar displayed parallel decreases in bacterial load when *R. rosea* fed flies were plated, demonstrating the similarities between the bacteria that are culturable when utilizing the non-selective nature of the nutrient media. A significant decrease in the CFUs with 10 days old flies fed *R. rosea* on MRS also suggests that *Lactobacillus* is responsible for variation in culturable bacteria at the earlier stages of the fly lifespan (Fig. 4a).

Previous reports demonstrated the impact of an altered diet such as changes in the sugar versus protein composition in the fly media affects the *Acetobacter* to *Lactobacillus* ratio in flies [52, 66]. Since a change in the diet impacts host physiology, the effects of the diet on the microbial community suggest that the health of the host is a major determinant for shaping the gut microbial population in flies [67]. Although studies have reported that the commensal bacterial load fluctuates throughout the *Drosophila* lifespan, we observed the dominance of *Acetobacter* throughout all stages of the fly life in both control and *R. rosea* fed flies [52]. Since *Acetobacter* species thrive under fully aerobic conditions and *Lactobacillus* species are incapable of thriving in a ubiquitously oxygenated environment, we propose the possibility that the gut oxygen tension experiences a shift towards aerobic conditions after supplementation of *R. rosea*, thus promoting the growth of *Acetobacter* [32, 68]. This is particularly more likely in older flies who consume more oxygen and produce a more severe physiological response to conventional oxygen intake when compared to their younger counterparts [69]. In addition to *R. rosea* playing a role in changing gut oxygen tension, we suspect the extract may further modify immune system function in the *Drosophila* gut. An altered gut microbial composition, as a result of the supplementation of *R. rosea,* may contribute towards limiting age-related dysplastic changes by positively modulating the process of mis-differentiation in intestinal stem cells (ISCs) and their progeny, leading to the improvement in intestinal function and subsequently benefitting the health of the host. Because epithelial barrier dysfunction is strongly associated with fly aging and mortality, we believe *R.* rosea may attenuate this process at the later stages of the fly life [70]. In summary, evaluating the impact of anti-aging botanical extracts, such as *R. rosea,* on the gut microbiome using *D.* melanogaster as a model system may provide a platform to understand the interactions between the microbiome, lifespan, and healthspan.

## Conclusions

This study demonstrates the effectiveness of using *D. melanogaster* as a model to study the effect of anti-aging botanical extracts on the gut microbial community. We observed changes in the relative abundance of order *Lactobacillales* and genus *Acetobacter* in the female fly lifespan. We also saw an increase in total bacterial load, and a decrease in OTU and CFU counts with supplementation of *R. rosea.* Future studies are needed to evaluate a potential link between major gut immune genes to bacterial diversity and abundance in order to thoroughly understand whether certain botanical extracts increase lifespan and improve healthspan by altering the gut microbiome.

## Additional files

**Additional file 1: Figure S1.** qRT-PCR and CFU analysis of male *D. melanogaster* at early and late stages of the fly lifespan.

**Additional file 2: Table S1.** OTU table provided by 16S rRNA amplicon sequencing of female *D. melanogaster* at early and late stages of the fly lifespan.

## Abbreviations

PCA: principal components analysis
qRT-PCR: quantitative real-time PCR
MRS: De Man, Rogosa and Sharpe
CFUs: colony forming units
OTU: operational taxonomic units
Ct: threshold cycle
ISCs: intestinal stem cells
