# Supplementary figures and images for: The impact of Rhodiola rosea on the gut microbial community of Drosophila melanogaster
Source: Gut Pathog. 2018 Mar 20;10:12. doi: 10.1186/s13099-018-0239-8 (PMC5861609; doi:10.1186/s13099-018-0239-8)

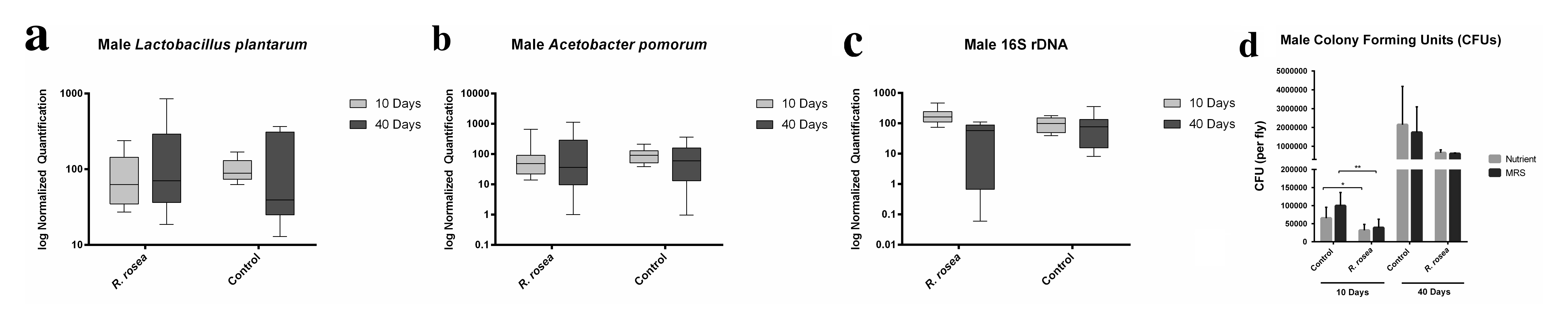

Supplement: Supplementary file 1 — Additional file 1: Figure S1. qRT-PCR and CFU analysis of male D. melanogaster at early and late stages of the fly lifespan. [file 13099_2018_239_MOESM1_ESM.png]
